# Supplementary material for: Causes and consequences of sex-chromosome turnovers in Diptera
Source: Evol Lett. 2026 Mar 12;10(3):253–68. doi: 10.1093/evlett/qrag003 (PMC13231274; doi:10.1093/evlett/qrag003)
Supplement: qrag003_Supplemental_Files [file qrag003_supplemental_files.zip › Supplementary_Datasets.zip!/Supplementary_Datasets/Dataset_2_Graphical_representation_outgroups/Tachinidae_Nowickia_ferox_chromosomes.pdf]

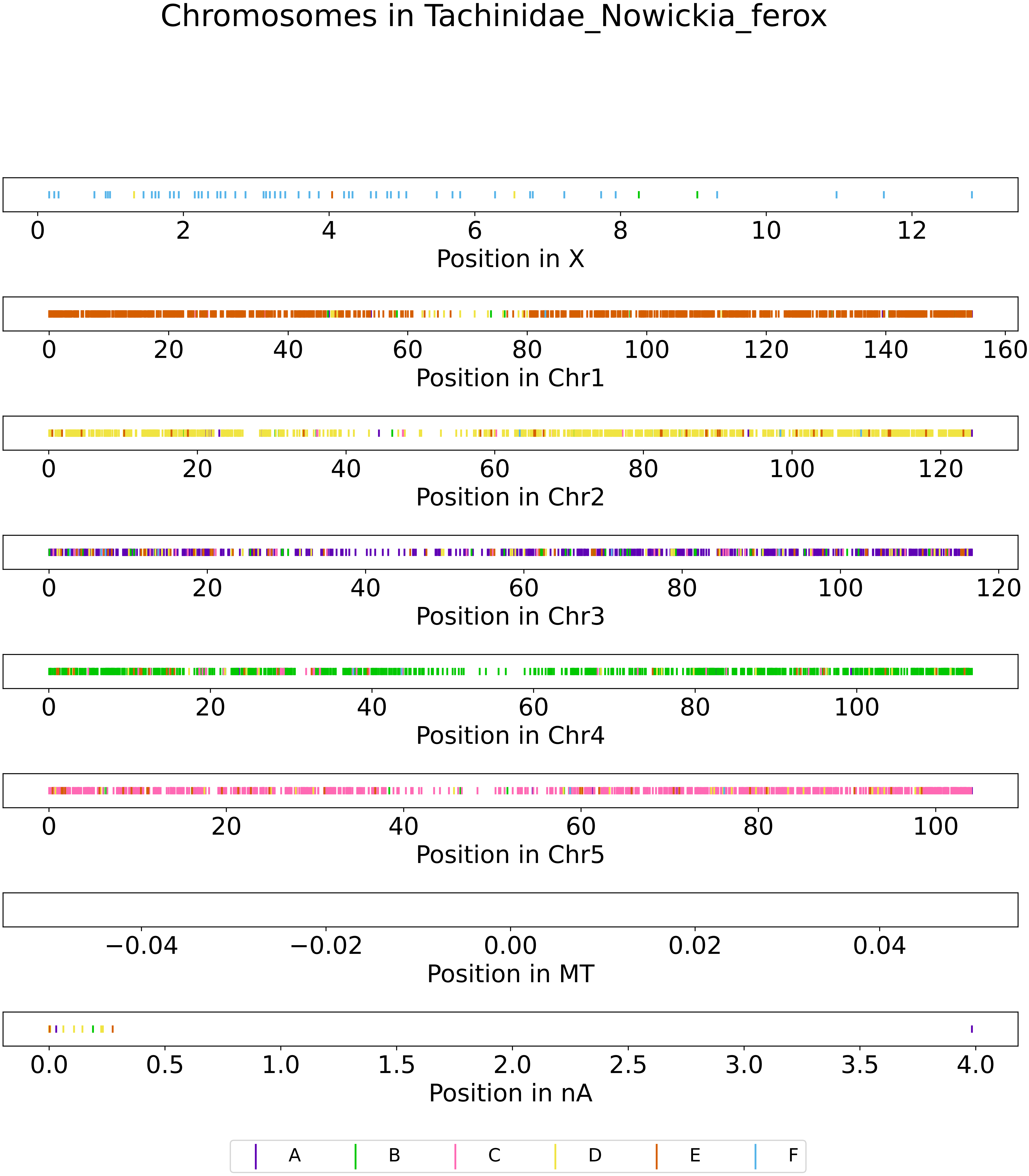

Chromosomes in Tachinidae_Nowickia_ferox
0
2
4
6
8
10
12
Position in X
0
20
40
60
80
100
120
140
160
Position in Chr1
0
20
40
60
80
100
120
Position in Chr2
0
20
40
60
80
100
120
Position in Chr3
0
20
40
60
80
100
Position in Chr4
0
20
40
60
80
100
Position in Chr5
−0.04
−0.02
0.00
0.02
0.04
Position in MT
0.0
0.5
1.0
1.5
2.0
2.5
3.0
3.5
4.0
Position in nA
A
B
C
D
E
F
